# Gluten Contamination of Labelled Gluten-Free Food Products Marketed in China

**DOI:** 10.3390/foods14122025

**Published:** 2025-06-08

**Authors:** Yanjun Li, Qing Liu, Samuel Godefroy, Jingguang Li, Yan Chen

**Affiliations:** 1 Research Unit of Food Safety, Chinese Academy of Medical Sciences (No. 2019RU014), NHC Key Laboratory of Food Safety Risk Assessment, China National Center for Food Safety Risk Assessment, Beijing 100021, China; liyanjun@cfsa.net.cn (Y.L.); liuqing@cfsa.net.cn (Q.L.); lijg@cfsa.net.cn (J.L.); 2 Food Risk Analysis and Regulatory Excellence Platform (PARERA), Institute of Nutrition and Functional Foods, Laval University, Quebec, QC G1V 0A6, Canada; samuel.godefroy@fsaa.ulaval.ca

**Keywords:** coeliac disease, food contamination, gluten, gluten-free diet, gluten-related disorders, labelled gluten-free, gluten quantification, ELISA

## Abstract

Gluten has a central role in the pathogenesis of several gluten-related disorders, with coeliac disease being one of the most well-known. A rigorous gluten-free diet is recognized as the only safe and efficient coeliac disease therapy. This study intended to use the enzyme-linked immunosorbent assay (ELISA) R5 Méndez method to determine gluten contamination in labelled gluten-free food products marketed in China and to evaluate the performance of two ELISA platforms (the Neogen Veratox^®^ R5 and the Romer Labs AgraQuant^®^ G12) for analyzing gluten, using R5 Méndez as the reference. In 2024, 119 prepackaged products labelled as gluten-free were purchased from internet suppliers. The results showed that among the 119 products, 13.4% contained gluten exceeding 20 mg/kg, and 5.0% contained over 100 mg/kg, ranging from 333.1 to 2737.4 mg/kg. When the threshold for gluten-free products was 20 mg/kg, the two ELISA platforms yielded results comparable to R5 Méndez. However, when the threshold was 10 mg/kg and 5 mg/kg, the McNemar *x*^2^ test showed significant differences between the proportions of positive results of the two ELISA platforms and R5 Méndez (*p*-value < 0.05); using R5 Méndez as the reference, the two ELISA platforms showed 53.1% to 70.3% positive predictive values, suffering the drawback of high false-positive results. When using stricter gluten limits of ≤10 mg/kg and ≤5 mg/kg, different ELISA platforms may produce different results for the same food product. These findings highlight the need for China to accelerate the development of national standards for gluten-free foods, and for food regulatory agencies to impose requirements of production that lead to the prevention of gluten contamination, confirmed by the possible monitoring of such compliance through routine sampling and testing. Such measures will no doubt contribute to protecting and improving the health of patients with gluten-related disorders.

## 1. Introduction

Gluten is the major structural protein found in grains such as wheat, barley, rye, and oats, and it has a central role in the pathogenesis of several gluten-related disorders (GRDs), including coeliac disease (CD), dermatitis herpetiformis, gluten ataxia, IgE-mediated wheat allergy, and non-coeliac gluten sensitivity [1]. CD is one of the most well-known GRDs, affecting approximately 1% of the global population [2]. A rigorous gluten-free diet (GFD) is recognized as the only safe and efficient CD therapy [3].

For most patients with CD, extremely low doses of gluten are tolerable. Evidence exists suggesting that exposure to less than 10 mg per day is unlikely to cause histological changes to the intestinal mucosa, while exposure to 50 mg per day is likely to do so [4,5,6]. These levels contributed to establishing that it is safe to consume gluten-free foods with a gluten content below 20 mg/kg [7]. This threshold has been adopted by major food regulatory agencies, including the Codex Alimentarius Commission [8], the European Food Safety Authority [9], and the United States Food and Drug Administration [10]. However, some organizations, such as the Gluten-Free Certification Organization (GFCO) in North America and the American Celiac Sprue Association (CSA), recommend even stricter gluten limits of ≤10 mg/kg and ≤5 mg/kg, respectively [11,12].

There are unique challenges within the Chinese market. Due to the insufficient research and understanding of GRDs, limited gluten control technology in enterprises, and a lack of effective localized gluten detection techniques, the formulation of a national standard may face regulatory difficulties. There is currently no national standard for gluten-free foods in China, and developing standards may face technical and regulatory difficulties. However, as a result of the lack of national standards, gluten-free foods in China are completely unregulated, posing health risks for patients with GRDs. To this end, the China National Center for Food Safety Risk Assessment (CFSA) launched the development of a national standard for gluten-free foods in 2023. CFSA’s research shows that based on the dietary habits of the Chinese population, if gluten levels do not exceed a maximum threshold of 20 mg/kg, even if a patient with CD consumes a large amount of gluten-free food, it would be very difficult to exceed the safe threshold of 10–50 mg of gluten per day [13].

Despite the increasing availability of naturally gluten-free and commercially produced gluten-free foods, completely eliminating gluten from the diet remains highly challenging. Gluten is one of the most widely used food ingredients in the food industry. The contamination of gluten-free foods with gluten-containing foods can occur at multiple stages of food production, from farms and factories to restaurants and homes [14,15]. The prolonged intake of gluten-contaminated foods may cause persistent intestinal damage and symptoms in patients with CD on a GFD [16].

Consumers rely on labels to choose gluten-free foods. However, reports from multiple countries indicate that gluten contamination is common in gluten-free foods [15]. Such data are not available in the case of China. At present, enzyme-linked immunosorbent assay (ELISA) is recommended by the Codex Alimentarius Commission as the analytical technique to realize gluten-free food labelling [17]. The differences in the results of certain food matrices by different test methods may lead to trade disputes or regulatory disagreements [18]. In 2006, the Codex Committee on Methods of Analysis and Sampling endorsed the ELISA R5 Méndez as a type I method for the determination of gluten content in gluten-free foods [19].

There is a lack of studies on gluten contamination in foods labelled as gluten-free marketed in China, as well as a lack of confirmatory research on ELISA platforms for analyzing gluten using various foods on the market. Therefore, this study aimed to use R5 Méndez to determine the degree of gluten contamination in labelled gluten-free food products available on the Chinese market and to evaluate the performance of two ELISA platforms (the Neogen Veratox^®^ R5 and the Romer Labs AgraQuant^®^ G12) for analyzing gluten, using R5 Méndez as the reference.

## 2. Materials and Methods

### 2.1. Food Products

In March 2024, 119 prepackaged food products labelled as gluten-free were purchased from Chinese internet suppliers. Different product types and brands were selected to rule out selection bias as much as possible. All products were assigned an individual laboratory number, and their detailed information (such as brand, production location, basic ingredients, and food category) was documented on an Excel spreadsheet. According to the basic ingredients of the food, products were classified as rice, mixed grains, legumes and potatoes, mixed food, oats, millet, corn, buckwheat, and quinoa. Mixed grains refer to foods with two or more grains as main ingredients. Mixed food refers to food that contains two or more different categories of ingredients, such as rice and soya. According to the food category, products were classified as flour, noodles, seasoning, biscuit and bread, snacks, and breakfast porridge. In total, 33 (27.7%) out of the 119 products were produced outside China, especially in Thailand (11), the United States (5), Australia, Germany (4 each), the United Kingdom (3), Spain (2), as well as Italy, New Zealand, the Philippines, and Sri Lanka (1 each). This study protocol is exempt from approval by the ethics committee of CFSA.

### 2.2. Determination of Gluten

Experiments were conducted centrally at CFSA. All reagents were of analytical grade. Three groups of personnel independently used different ELISA platforms to detect gluten in products. The manufacturer’s instructions were strictly adhered to for each analysis.

#### 2.2.1. Sample Extraction and Preparation

Samples were homogenized well with a sufficient amount (50 g or 50 mL of each food sample) to ensure taking a representative test portion. Homogenized samples were stored in centrifuge tubes, with at least 5 g of processed sample in each tube. To prevent potential cross-contamination, samples were homogenized at different times and in different rooms. For each sample processed, the blender and the environmental surfaces were rinsed with 60% ethanol. For every 10 products, the gluten contamination on environmental surfaces was determined using RIDA^®^QUICK Gliadin immunochromatographic strips (R7003) from R-Biopharm (Darmstadt, Germany). No environmental gluten contamination was observed during testing, implicating the validity of the analytical data.

#### 2.2.2. Gluten Quantification

Using the cocktail solution (patented) (R7006) from R-Biopharm (Darmstadt, Germany) to extract gluten and RIDASCREEN^®^ Gliadin sandwich R5 ELISA (R7001) from R-Biopharm to determine the gluten content, this combination was defined as the ELISA R5 Méndez method. The Veratox^®^ for Gliadin R5 test kit (Neogen, Lansing, MI, USA) and the AgraQuant^®^ Gluten G12 test kit (Romer Labs, Getzersdorf, Austria) were also used for quantitative analysis. The characteristics of the three commercial sandwich ELISA test kits used in this study to analyze gluten in different food products are shown in Table 1. In this study, we utilized the RIDASCREEN^®^ Gliadin sandwich R5 ELISA, which detects intact gliadins and related prolamins from rye and barley, high-molecular-weight glutenin subunits from wheat, high-molecular-weight secalins from rye, and low-molecular-weight glutenin subunits from wheat. However, this method does not detect D-hordeins from barley [20]. Combined with the cocktail (patented), it has been endorsed as a Codex Alimentarius Type I method for the analysis of gluten [19] and has been adopted by AOAC INTERNATIONAL as the Final Action Official Method^SM^ 2012.01 with an “in foods” claim [21]. Due to the inaccurate quantification of hydrolyzed gluten by sandwich ELISA, the RIDASCREEN^®^ Gliadin competitive R5 ELISA (R7021) from R-Biopharm (Darmstadt, Germany) was used to screen products known to have been subjected to hydrolysis in order to detect smaller gluten peptide fragments that may escape the detection of the sandwich ELISA.

### 2.3. Statistical Analysis

The results are expressed as incidences, medians and ranges, means, and standard deviations (SD) as appropriate. Fisher‘s exact test was used to compare the proportion of contaminated (>20 mg/kg) and uncontaminated (≤20 mg/kg) products in each group. The McNemar *x*^2^ test was used to compare the proportion of positive results, and Kappa statistics were used to measure the agreement degree (Kappa coefficient) between the two ELISA platforms and R5 Méndez, which evaluated the same products. *K*-values of > 0.75 represented excellent agreement, and values of 0.40 to 0.75 represented moderate-to-good agreement [22]. Method performance characteristics, including sensitivity, specificity, and the predictive value of positive and negative results, were investigated for several preselected gluten-free thresholds and were calculated as described [23]. The level of significance was a *p*-value < 0.05. Statistical analyses were performed using SPSS for Windows Release 16.0 program (SPSS Inc., Chicago, IL, USA).

## 3. Results

A total of 119 food products were collected, all of which were investigated for gluten contamination using the R5 Méndez. According to the mean gluten concentration of 2 extractions, 91 (76.5%) out of the 119 products contained less than 5 mg/kg of gluten, 7 (5.9%) contained between 5 and 10 mg/kg, 5 (4.2%) contained between 10 and 20 mg/kg, and 16 (13.4%) contained more than 20 mg/kg (ranging from 28.4 to 2737.4 mg/kg) (Table 2). In total, 6 out of the 16 gluten-contaminated products (37.5%) had levels above 100 mg/kg, ranging from 333.1 to 2737.4 mg/kg.

Among the 33 imported products, 31 (93.9%) contained less than 5 mg/kg of gluten, 1 (3.0%) contained between 5 and 10 mg/kg, 1 (3.0%) contained between 10 and 20 mg/kg, and none contained more than 20 mg/kg. Among the 86 domestically made products, 60 (69.8%) contained less than 5 mg/kg of gluten, 6 (7.0%) contained between 5 and 10 mg/kg, 4 (4.7%) contained between 10 and 20 mg/kg, and 16 (18.6%) contained more than 20 mg/kg. There was a significant difference in the prevalence of gluten contamination between imported and domestically made gluten-free products (*p*-value < 0.01).

The gluten contamination in labelled gluten-free products based on the basic ingredients and food category is shown in Table 3 and Table 4, respectively. Significant differences (*p*-value < 0.05) were found in the proportion of gluten contamination in food products with different basic ingredients, with higher proportions of contamination in buckwheat (50.0%), quinoa (50.0%), millet (33.3%), mixed grain (25.0%), and corn (20%) products, whereas gluten was not detected in oats and mixed foods (Table 3). By food category, flour (23.1%) and biscuit and bread (18.8%) had higher proportions of contamination than seasonings (4.5%), but the difference was not statistically significant (*p*-value > 0.05) (Table 4).

A total of 22 seasoning products were tested: 19 liquids and 3 solids. All liquid products contained less than 5 mg/kg of gluten. Among the three solid seasoning products, one (33.3%) contained between 10 and 20 mg/kg, and one (33.3%) contained more than 20 mg/kg of gluten.

Eighteen products are known to have undergone hydrolysis treatment, including seventeen liquid seasoning products and one bread product, all of which contained gluten below the limit of quantification (LOQ) (5 mg/kg), as determined by R5 Méndez. These products were retested using the competitive R5 ELISA, and the gluten content of all products was below the LOQ of the method (10 mg/kg), which means that after confirmation, all product results remained negative.

When the threshold for gluten-free products was 20 mg/kg, the McNemar test showed no significant difference between the two ELISA platforms and R5 Méndez (*p*-value > 0.05), and Kappa statistics showed excellent agreement (*K*-value > 0.75, *p*-value < 0.001) (Table 5). Taking R5 Méndez as the reference, Veratox^®^ for Gliadin R5 showed 100.0% sensitivity, 98.1% specificity, and the predictive values of positive and negative results of 88.9 and 100.0%, respectively; AgraQuant^®^ Gluten G12 showed 100.0% sensitivity, 96.1% specificity, and the predictive values of positive and negative results of 80.0 and 100.0%, respectively (Table 6).

When the threshold was 10 mg/kg and 5 mg/kg, fair-to-good agreement was observed between the two ELISA platforms and R5 Méndez (*K*-value between 0.40 and 0.75, *p*-value < 0.001). However, a significant difference in the proportion of positive results by the two ELISA platforms was observed in the McNemar test (*p*-value < 0.05) (Table 5). Taking R5 Méndez as the reference, the two ELISA platforms showed 90.5% to 95.2% sensitivity, 74.7% to 90.8% specificity, 53.1% to 70.3% positive predictive value, and 97.1% to 98.9% negative predictive value but suffered from the drawback of high false-positive results (Table 6).

Of the 119 products tested, 16 were detected to have gluten above 20 mg/kg by R5 Méndez (13.4%), 18 by Veratox^®^ for Gliadin R5 (15.1%), and 19 by AgraQuant^®^ Gluten G12 (16.0%). Sixteen products (13.4%) tested positive in all three platforms employed. The concentrations of gluten in contaminated (>20 mg/kg) products detected using the three types of sandwich ELISA platforms are shown in Table 7.

## 4. Discussion

To our knowledge, this is the first report on gluten contamination in foods labelled as gluten-free in China. Our findings indicate that gluten contamination in gluten-free products sold through Chinese internet suppliers is common, as reflected by both the proportion (13.4%) and the gluten level (37.5% of the contaminated products had a gluten level above 100 mg/kg) of the contaminated food products.

Most of the recent studies on gluten contamination have been conducted using the same R5 Méndez, which helps to compare findings obtained from different studies [24]. The findings of the present study are consistent with the global trends when compared to the contamination studies of labelled gluten-free foods conducted in other countries [15,24]. In a meta-analysis that included 40 articles, the results showed that in the labelled gluten-free foods analyzed, the prevalence of gluten contamination was estimated at 9.5% (95% CI: 4.8%, 15.7%) [24]. Gluten levels above 20 mg/kg were detected in 2.2% (2/98) of the products labelled as gluten-free in Italy [25]. In Canada, gluten levels above 20 mg/kg were found in 7 (9.1%) out of the 77 cereal foods labelled as gluten-free [26]. In a study conducted in southern India, 5 (9.8%) out of 51 grain products labelled as gluten-free showed gluten contamination above 20 mg/kg, although the levels fell within the low gluten range (32.5 ± 5.8) [27]. Gluten contamination was found in 11.5% of labelled gluten-free foods on the Turkish market [28]. In the United States, 16 (20.5%) out of the 78 products labelled as gluten-free foods were found to contain ≥20 mg/kg of gluten, with levels ranging from 20.3 to 60.3 mg/kg [29]. According to Valdés et al., 1071 out of 3088 (34.7%) gluten-free foods in Europe had gluten contamination above 20 mg/kg [30].

It is worth noting that none of the imported products contained gluten above 20 mg/kg, while 16 out of 86 (18.6%) Chinese-made products did, indicating potential regulatory or production practice differences that might explain this discrepancy. The current situation suggests that there is less attention paid to attaining a safe level of gluten in products meant for patients with GRDs and labelled as gluten-free when produced domestically. This represents a significant risk to the food safety of patients with GRDs, as unintentional gluten exposure can have serious health consequences.

Consistent with previous studies, we found a higher risk of gluten contamination in buckwheat (2/4) and quinoa (1/2) products labelled as gluten-free on the Chinese market [25,29,31]. Specific matrices like buckwheat, quinoa, and millet had higher contamination levels, indicating the possibility of cross-contamination between these natural gluten-free grains and gluten-containing grains during planting and processing and the need to strengthen the regulation of such natural gluten-free grains. However, unlike studies conducted in other countries, we did not detect gluten contamination in oat products [24]. Previous research has shown that cross-contamination is particularly prevalent in oats that are not specifically produced and labelled as gluten-free. This difference may be explained by the fact that the oats tested in this study were all marked or identified as gluten-free and therefore must have followed stricter controls to prevent the introduction of foreign gluten-containing grains. Several studies have shown that patients with CD can safely consume moderate amounts of oats that are not contaminated with gluten [32,33]. However, commercial oat supplies are frequently contaminated with wheat. For instance, a study in Canada found that 88% of 133 oat products contained more than 20 mg/kg of gluten [34]. The cross-contamination of naturally gluten-free foods can occur at various points in the food chain, including at planting, harvesting, and/or processing. If naturally gluten-free grains are rotated with wheat, barley, rye, or oats, the mixing of grains in the field can occur [15]. Additionally, gluten-containing seeds can persist in the soil, leading to the unintentional collection of gluten-containing grains during harvest. Further contamination may occur when storage facilities and transport systems are shared. Moreover, food manufacturers often use the same equipment and facilities for producing both gluten-free and gluten-containing products, further increasing the risk of cross-contamination.

The proportion of contaminated flour-based products in this study exceeded 20%. One of the products was rye flour with a gluten contamination concentration of 56.7 mg/kg. The product claimed to be gluten-free and had posted a report on its website stating that no gluten was detected, but it did not specify whether gluten was removed during the product processing. Rye itself contains gluten, and retailers classify products as gluten-free based on test results, indicating an urgent need for the standardization of gluten testing methods by third-party testing companies.

The measurement error between different commercial ELISA test kits for the quantification of gluten content is significant [17,18,35]. The assessment of several commercial ELISA test kits against a reference method for gluten analysis is of general importance, especially for the assessment of targeted gluten-free threshold limits, which were preselected based on the currently valid regulations or limits considered/newly proposed in the current scientific literature. In the present study, when the threshold for gluten-free products was 20 mg/kg, the two ELISA platforms under study yielded results comparable to R5 Méndez. However, when the threshold was 10 mg/kg and 5 mg/kg, a significant difference was observed between the proportions of positive results from the two ELISA platforms and R5 Méndez. Due to the relatively low positive predictive values of the two ELISA platforms, it is necessary to address the possibility of missing products with gluten levels above the threshold, as this will increase the management costs of the enterprise. The differences in test results caused by different ELISA platforms may also lead to regulatory issues, especially regarding policy or trade disputes that may arise from inconsistent gluten quantification at lower thresholds.

Currently, there is a lack of education about gluten-free foods throughout Chinese society, and the concept of gluten is extremely vague to consumers [36]. A meta-analysis revealed that the prevalence of gluten contamination in labelled products offered by food services was 41.5% (95% CI: 16.6%, 66.4%), and the probability of obtaining gluten-contaminated gluten-free food in food services is higher than at home [37]. Due to the lack of a national standard for gluten-free foods, Chinese restaurant workers receive insufficient systematic training, and training and education policies regarding gluten-free dietary requirements should be actively pursued for food service employees. Patients with GRDs must be cautious when checking labels and learning about food preparation while dining outdoors. Such measures will no doubt contribute to protecting and improving the health of patients with GRDs.

The results highlight the complexity of maintaining a GFD. However, due to issues related to labelling, cross-contamination, and trace amounts of gluten, patients with GRDs face unique challenges. Strict adherence to a GFD is crucial for these individuals, but the aforementioned issues can impede proper compliance. Inaccurate labelling or inadequate standards may result in unintentional gluten exposure, while cross-contamination and trace amounts of gluten present ongoing risks. Therefore, it is essential to explore additional measures needed to ensure food safety for this group, including stricter regulations and clearer labelling practices to minimize contamination risks and ensure that gluten-free products meet the required standards.

To ensure the long-term health and food safety of patients with GRDs, it is essential for Chinese food regulatory agencies to establish comprehensive national standards for gluten-free foods. This could benefit from more concrete recommendations on implementation, such as the structure of a Chinese certification system in alignment with the framework of the Codex Alimentarius Commission/European Food Safety Authority. A certification foundation could be established for companies, similar to organizations in other countries, through laboratories accredited by recognized bodies and authorized to detect and quantify gluten. Certification bodies accredited under relevant standards for the agro-food industry would also be essential to ensure food safety. Additionally, an annual review of Hazard Analysis and Critical Control Point systems in the food industry should be conducted to ensure ongoing compliance and further minimize the risk of gluten contamination.

The main limitation of this study is the reliance on products from internet suppliers only, which may not represent the full domestic market. Another limitation is the quality assurance measures across the three ELISA platforms. This study mainly relies on independent personnel using different ELISA platforms to detect gluten in products without conducting inter-laboratory calibration or recovery studies to validate their comparative claims.

## 5. Conclusions

This study provides important insights into the status of gluten contamination in food products labelled as gluten-free on the Chinese market, addresses an important food safety issue in a growing consumer market, and has the potential to serve as a reference point for policy reform. Research has shown that gluten contamination in Chinese domestically produced gluten-free products is common, with some products containing relatively higher levels of gluten. We believe that all analytical platforms that can provide results comparable to R5 Méndez are applicable for gluten determination. When using stricter gluten limits of ≤ 10 mg/kg and ≤ 5 mg/kg, different ELISA platforms may produce different results for the same food product. These findings highlight the need for Chinese food regulatory agencies to impose requirements for production that lead to preventing gluten contamination, confirmed by the possible monitoring of such compliance through routine sampling and testing. Gluten-free food manufacturers need to confirm that raw materials do not contain gluten and eliminate cross-contamination during the food production stage. This can be remedied by establishing standards and stricter controls on the future labelling of gluten-free foods in China to ensure that the risk of gluten contamination is reduced for patients with GRDs. Our recommendations for improving gluten-free food regulation in China can also provide references for countries lacking regulatory measures for gluten-free foods.

## Figures and Tables

**Table 1:** Characteristics of commercial sandwich ELISA test kits.

Test Kit	Manufacturer	Antibody	Quantification Range (mg/kg Gluten)	Limit of Quantification (mg/kg Gluten)	Main Epitope
RIDASCREEN^®^ Gliadin Sandwich R5 ELISA	R-Biopharm	R5 mAb	5–80	5.0	QQPFP
Veratox^®^ for Gliadin R5	Neogen	R5 mAb	5–80	5.0	QQPFP
AgraQuant^®^ Gluten G12	Romer Labs	G12 mAb	4–200	4.0	QPQLPY

**Table 2:** Level of gluten contamination in the 119 analyzed products labelled as gluten-free using the ELISA R5 Méndez method.

Gluten Content (mg/kg)	Number of Products	Median (Range) (mg/kg)	Mean ± SD ^1^ (mg/kg)
5–10	7	7.1 (5.9–9.8)	7.4 ± 1.3
10–20	5	12.1 (10.3–17.3)	12.8 ± 2.7
>20	16	75.2 (28.4–2737.4)	384.2 ± 712.4

^1^ SD, standard deviation.

**Table 3:** Incidence and range of gluten contamination in the 119 analyzed products labelled as gluten-free by basic ingredients using the ELISA R5 Méndez method ^1^.

Basic Ingredient	No. of Products	Incidence (%) of <5 mg/kg Gluten	5–10 mg/kg		10–20 mg/kg		>20 mg/kg	
			Incidence (%)	Range (mg/kg)	Incidence (%)	Range (mg/kg)	Incidence (%)	Range (mg/kg)
Rice	36	29 (80.6)	2 (5.6)	5.9–8.0	3 (8.3)	10.3–17.3	2 (5.6)	35.9–390.1
Mixed grains	20	14 (70.0)	1 (5.0)	9.8	– ^2^	–	5 (25.0)	45.5–2737.4
Legumes and potatoes	17	14 (82.4)	2 (11.8)	7.1–7.7	–	–	1 (5.9)	47.8
Mixed food	10	8 (80.0)	2 (20.0)	6.3–6.6	–	–	–	–
Oats	7	7 (100.0)	–	–	–	–	–	–
Millet	6	4 (66.7)	–	–	–	–	2 (33.3)	75.2–1381.3
Corn	5	4 (80.0)	–	–	–	–	1 (20.0)	28.4
Buckwheat	4	1 (25.0)	–	–	1 (25.0)	11.5	2 (50.0)	331.1–335.3
Quinoa	2	1 (50.0)	–	–	–	–	1 (50.0)	64.9
Others	12	9 (75.0)	–	–	1 (8.3)	12.1	2 (16.7)	56.7–75.2
Total	119	91 (76.5)	7 (5.9)	5.9–9.8	5 (4.2)	10.3–17.3	16 (13.4)	28.4–2737.4

^1^ the incidence is the number of products containing the specified concentration of gluten. ^2^ none of the products contained the specified concentration of gluten.

**Table 4:** Incidence and range of gluten contamination in the 119 analyzed products labelled as gluten-free by food category using the ELISA R5 Méndez method ^1^.

Food Category	No. of Products	Incidence (%) of <5 mg/kg Gluten	5–10 mg/kg		10–20 mg/kg		>20 mg/kg	
			Incidence (%)	Range (mg/kg)	Incidence (%)	Range (mg/kg)	Incidence (%)	Range (mg/kg)
Flour	26	18 (69.2)	2 (7.7)	7.7–9.8	– ^2^	–	6 (23.1)	56.7–1381.3
Noodles	27	21 (77.8)	2 (7.4)	6.3–7.1	1 (3.7)	11.5	3 (11.1)	45.5–2737.4
Seasoning	22	20 (90.9)	–	–	1 (4.5)	12.1	1 (4.5)	75.2
Biscuit and bread	16	10 (62.5)	2 (12.5)	5.9–6.6	2 (12.5)	10.3–17.3	2 (12.5)	28.4–35.9
Snacks	16	11 (68.8)	1 (6.3)	8.0	1 (6.3)	12.8	3 (18.8)	61.4–76.2
Breakfast porridge	12	11 (91.7)	–	–	–	–	1 (8.3)	47.8
Total	119	91 (76.5)	7 (5.9)	5.9–9.8	5 (4.2)	10.3–17.3	16 (13.4)	28.4–2737.4

^1^ the incidence is the number of products containing the specified concentration of gluten. ^2^ none of the products contained the specified concentration of gluten.

**Table 5:** Results of the McNemar *x*^2^ test and Kappa statistics to check the equality and measurement of agreement between the observed outcome of the ELISA platforms under study, taking the ELISA R5 Méndez method as the reference.

Threshold for Gluten-Free Products (mg/kg)	ELISA Platforms	McNemar Test		Kappa Agreement	
		OR ^1^	Exact Sig. (2-Sided)	*K*-Value ^2^	Exact Sig. ^3^
20	Veratox^®^ for Gliadin R5	9.0	0.500	0.931	0.000
	AgraQuant^®^ Gluten G12	5.0	0.125	0.869	0.000
10	Veratox^®^ for Gliadin R5	197.8	0.021	0.749	0.000
	AgraQuant^®^ Gluten G12	48.7	0.001	0.588	0.000
5	Veratox^®^ for Gliadin R5	94.5	0.022	0.727	0.000
	AgraQuant^®^ Gluten G12	38.4	0.000	0.537	0.000

^1^ OR, odds ratio. ^2^ *K*-value, Kappa coefficient. ^3^  Sig., *p*-value.

**Table 6:** Performance comparison of the two ELISA platforms, taking the ELISA R5 Méndez method as the reference.

Threshold for Gluten-Free Products (mg/kg)	ELISA Platforms	Sensitivity	Specificity	Positive Predictive Value	Negative Predictive Value
20	Veratox^®^ for Gliadin R5	100.0	98.1	88.9	100.0
	AgraQuant^®^ Gluten G12	100.0	96.1	80.0	100.0
10	Veratox^®^ for Gliadin R5	95.2	90.8	69.0	98.9
	AgraQuant^®^ Gluten G12	90.5	83.7	54.3	97.6
5	Veratox^®^ for Gliadin R5	92.9	87.9	70.3	97.6
	AgraQuant^®^ Gluten G12	92.9	74.7	53.1	97.1

**Table 7:** Concentrations of gluten in contaminated (>20 mg/kg) products using three types of sandwich ELISA platforms.

Food Category	Basic Ingredient	ELISA R5 Méndez Method	Veratox^®^ for Gliadin R5	AgraQuant^®^ Gluten G12
Flour	Rice	>80	>80	>200
Flour	Millet	>80	>80	>200
Flour	Buckwheat	>80	>80	>200
Flour	Buckwheat	>80	>80	>200
Flour	Quinoa	64.9	>80	193.3
Flour	Others	56.7	>80	>200
Noodles	Mixed grains	45.5	>80	57.7
Noodles	Mixed grains	>80	>80	>200
Noodles	Mixed grains	>80	>80	>200
Noodles	Legumes and potatoes	7.1	22.8	15.1
Seasoning	Others	Below LOQ ^1^	Below LOQ	28.0
Seasoning	Others	75.2	>80	>200
Biscuit and bread	Rice	5.9	11.6	45.4
Biscuit and bread	Rice	17.3	27.8	22.1
Biscuit and bread	Rice	35.9	>80	86.3
Biscuit and bread	Corn	28.4	89.0	50.8
Snacks	Rice	12.8	16.3	43.4
Snacks	Millet	75.2	>80	>200
Snacks	Mixed grains	61.4	>80	158.6
Snacks	Mixed grains	76.2	>80	>200
Breakfast porridge	Legumes and potatoes	47.8	>80	155.2

^1^ LOQ, limit of quantification.

## Data Availability

The original contributions presented in the study are included in the article, further inquiries can be directed to the corresponding author.

## Abbreviations

The following abbreviations are used in this manuscript:

CD	Coeliac disease
CFSA	China National Center for Food Safety Risk Assessment
ELISA	Enzyme-linked immunosorbent assay
GFD	Gluten-free diet
GRDs	Gluten-related disorders
LOQ	Limit of quantification
